# Sperm capacitation and transcripts levels are altered by in vitro THC exposure

**DOI:** 10.1186/s12860-023-00468-3

**Published:** 2023-02-23

**Authors:** Vivien B. Truong, Ola S. Davis, Jade Gracey, Michael S. Neal, Jibran Y. Khokhar, Laura A. Favetta

**Affiliations:** 1 Reproductive Health and Biotechnology Lab, Department of Biomedical Sciences, Ontario Veterinary College, University of Guelph, 50 Stone Rd. East, N1G 2W1 Guelph, ON Canada; 2 ONE Fertility, Burlington, ON Canada; 3 Department of Anatomy and Cell Biology, Western University, London, ON Canada

**Keywords:** Cannabis, THC, Sperm, Capacitation, Transcriptome, Fertility, Motility, Morphology, Apoptosis

## Abstract

**Background:**

Delta-9-tetrahydrocannabinol (THC) is the primary phytocannabinoid responsible for the psychoactive properties of cannabis and is known to interact with the endocannabinoid system, which is functionally present in the male reproductive system. Since cannabis consumption is the highest among reproductive aged males, the current study aimed to further investigate the effects of THC exposure to phenotypical, physiological, and molecular parameters in sperm. Bull sperm of known fertility were used as a translational model for human sperm and subjected to in vitro treatment with physiologically relevant experimental doses of THC. Sperm parameters, capacitation, apoptosis, and transcript levels were evaluated following treatment.

**Results:**

Motility, morphology, and viability of bovine sperm was unaltered from THC exposure. However, 0.32µM of THC caused an increased proportion of capacitating sperm (p < 0.05) compared to control and vehicle group sperm. Transcriptome analysis revealed that 39 genes were found to be differentially expressed by 0.032µM THC exposure, 196 genes were differentially expressed by 0.32µM THC exposure, and 33 genes were differentially expressed by 3.2µM THC. Secondary analysis reveals pathways involving development, nucleosomes, ribosomes and translation, and cellular metabolism to be significantly enriched.

**Conclusion:**

Phytocannabinoid exposure to sperm may adversely affect sperm function by stimulating premature capacitation. These findings also show for the first time that spermatozoal transcripts may be altered by THC exposure. These results add to previous research demonstrating the molecular effects of cannabinoids on sperm and warrant further research into the effects of cannabis on male fertility.

**Supplementary Information:**

The online version contains supplementary material available at 10.1186/s12860-023-00468-3.

## Introduction

An estimated 50 million couples are affected by infertility globally, a population that continues to grow [1]. Male factor infertility is suggested to account for 20% of instances of infertility and plays a role in another 30% of cases [2]. Since natural biological causes of infertility cannot account for all cases, environmental and lifestyle variables, including obesity, exposure to endocrine disrupting compounds, cigarette smoking, drug and alcohol use have gained significant attention in fertility studies.

Cannabis is recognized to be the most widely consumed recreational substance globally, with over 6.4 million Canadians and 48.2 million Americans having reported cannabis use in 2019 alone [3, 4]. It is known that men consume more cannabis compared to women and are also more likely to consume higher potency products [3, 5–7]. The overall high prevalence of cannabis use in recent years coincides with legislative changes permitting therapeutic cannabis, such as Nabilone and Dronabinol, which have been prescribed for anti-emetic purposes [8]. Cannabinoids have also been used to alleviate spasticity in multiple sclerosis patients and have been shown to have anti-anxiolytic, analgesic, and anti-epileptic properties [9, 10]. Medicinal cannabis use in the US alone increased over four-fold between 2016 and 2020 based on medicinal cannabis program enrollment [11]. Continual support of legalization and increasing social acceptability of cannabis use raise concerns about how cannabis may impact fertility and reproductive health, especially among reproductive aged men [7].

Delta-9-tetrahydrocannabinol (THC) is the phytocannabinoid responsible for inducing the psychoactive effects of cannabis, including euphoric feelings and cognitive impairment [12]. While delta-9-tetrahydrocannabinol is the primary psychoactive cannabinoid naturally derived from cannabis sativa, several other isoforms of THC have been identified including delta-8-tetrahydrocannabinol which contributes to approximately 10% of THC content in cannabis [13, 14]. Other major isoforms of THC include delta-10-tetrahydrocannabinol and delta-6a(10a)-tetrahydrocannabinol, which are synthetic isomers [15]. Furthermore, levels of THC found in cannabis continues to trend upwards, due to selective breeding of the cannabis plant to cater to consumer preference for higher potency products [16, 17]. The use of *THC* in the remaining text of this paper will refer to delta-9-tetrahydrocannabinol.

THC can activate cannabinoid receptors 1 and 2 (CB1/2), belonging to the body’s endocannabinoid system (ECS) [18]. The ECS is a pro-homeostatic system of receptors, enzymes, and ligands coined as endocannabinoids (eCBs) [18, 19]. Anandamide (AEA) and 2-arachidonylglycerol (2-AG) are the two endogenously produced cannabinoids, coined as endocannabinoids (eCBs), belonging to this system [18, 20]. The ECS was first identified in the central nervous system, but has since been confirmed to be present in the male and female reproductive systems [21, 22]. Functional cannabinoid receptors and eCBs present in mammalian sperm play a significant role in modulating sperm motility, viability, and capacitation [21, 23, 24]. In this regard, THC behaves as an exocannabinoid, stimulating CB1 and CB2 as a partial agonist [25].

The working and dynamic relationship between the ECS and sperm function has been successfully studied. In humans, eCB levels in seminal fluid were found to be inversely associated with fertility [21]. These results were corroborated by the work of Amoako et al. [26] who reported reduced seminal AEA levels observed among asthenozoospermic and oligoasthenoteratozoospermic men. Furthermore, stimulation of either cannabinoid receptors has been found to negatively impact sperm motility and viability in human sperm and inhibit capacitation and the acrosome reaction in boar sperm [23, 26–28].

Observational studies conducted among cannabis users also show detrimental effects of cannabis on sperm parameters, sperm epigenetics, and molecular and behavioural endpoints in offspring. Most notably, several studies report lower sperm concentrations among cannabis smokers and some suggesting that THC specifically can negatively impact spermatogenesis [29–32]. Cannabis users are also more likely to exhibit abnormal sperm morphology, a traditional indication of sperm quality and fertility [33–36]. Altered methylation patterns have also been observed in sperm from humans and rodents exposed to cannabis [32, 37]. Several of these methylation changes were also identified in the offspring of male mice exposed to cannabis during the preconception period [38]. Paternal cannabis exposure in rodents has also been associated with long-term neurological and behavioural differences in offspring [39, 40].

Despite the number of existing studies regarding the role of the ECS in sperm and observational studies on cannabis users, there have been limited explorations on the direct effect of THC to sperm. Whan et al. [41] explored the effects of THC on fresh human sperm by using concentrations of THC ranging from 0.032µM to 4.8µM, representing levels of THC found after therapeutic use of Marinol, an FDA approved cannabis product, and recreational cannabis use [42]. Both progressive sperm motility and spontaneous acrosome reaction were found to be inhibited by THC in a dose-dependent manner [41].

There have also been limited investigations on changes to sperm transcripts in response to exogenous agents, despite their abundance and evidence of differential gene expression between fertile and infertile sperm [43–45]. In addition, an essential subset of early embryonic transcripts is known to be exclusively derived from sperm [46].

In this study we aimed to evaluate the effects of delta-9-tetrahydrocannabinol (THC), the dominant naturally occurring isomer, on sperm motility and morphology as traditional indicators of sperm quality, in addition to viability and capacitation, which reflect physiological competence [35, 47–49]. Lastly, we explored the potential effects of THC on sperm transcriptome as sperm transcripts are reflective of fertility [43–45]. We hypothesized that THC would adversely affect motility, morphology, and viability, inhibit capacitation, and alter transcript levels. Owing to the constrains of human sperm use, the bovine model was chosen in the present study for its morphological and physiological similarity to human sperm [50, 51].

## Results

### Effects of THC on motility and morphology

A total of four biological replicates were assessed for both motility and morphology following THC treatment for 6- and 12- hours, representing individual ejaculates from four bulls. These treatment lengths were determined by initial motility and morphology experiments after treatment for various timepoints (Additional File 1, 2).

**Fig. 1:**
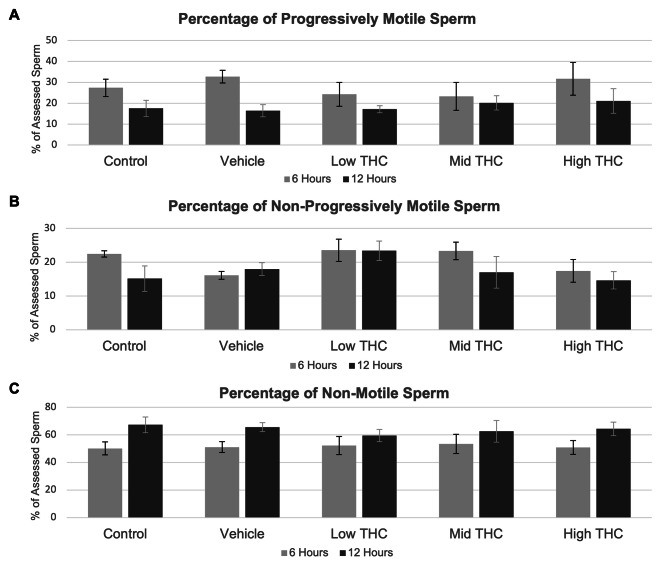
Motility characteristics following THC-treatment. Effect of therapeutic (low THC; 0.032 µM THC) and recreational (mid and high THC; 0.32 µM THC and 3.2 µM THC, respectively) concentrations of THC on (A) percentage progressive motility, (B) percentage non-progressive motility, and (C) percentage non-motile sperm. Motility was determined by manual sperm assessment of a minimum of 100 sperm per replicate after 6 and 12 h of exposure using a Makler counting chamber. Error bars represent average ± SEM, n = 4

**Fig. 2:**
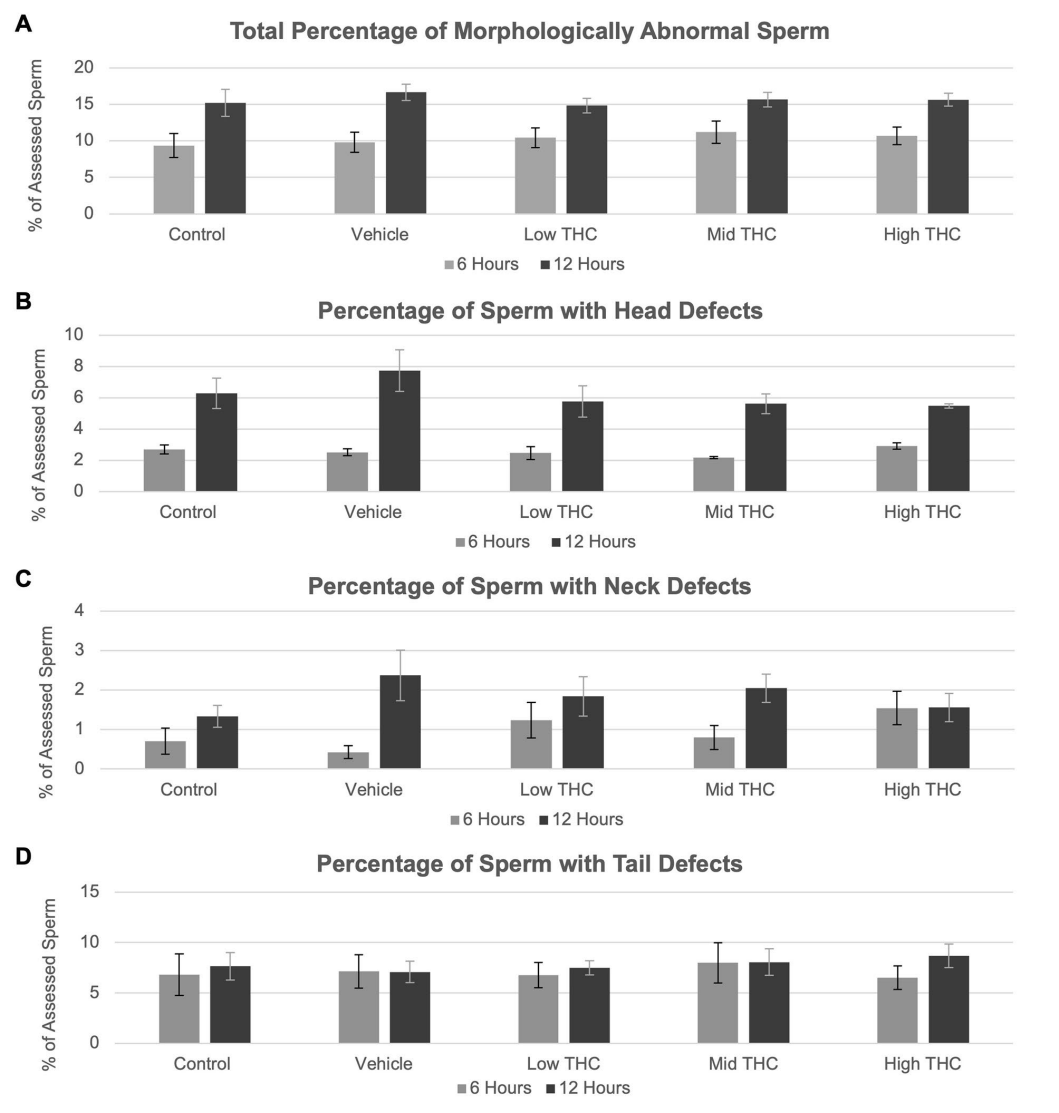
Morphology characteristics following THC-treatment. Effect of therapeutic (low THC; 0.032 µM THC) and recreational (mid and high THC; 0.32 µM THC and 3.2 µM THC, respectively) concentrations of THC on (A) overall sperm morphology, (B) head defects, (C) neck defects, and (D) tail defects. Morphology was manually determined using a minimum of 200 sperm per group per replicate after 6 and 12 h of treatment. Error bars represent ± SEM, n = 4

Sperm were categorized based on progressive motility, non-progressive motility, and non-motile. As shown in Fig. 1A-C, THC exposure for 6- or 12-hours at any concentration did not significantly affect motility. THC-treated sperm did not have significantly altered sperm morphology when evaluated as total percentage of cumulative abnormal sperm assessed or as total percentage of assessed sperm with head, neck, or tail defects (Fig. 2). Baseline sperm characteristics of the bulls used in subsequent motility and morphology experiments can be found in Additional File 3.

### Effects of THC on sperm viability

**Fig. 3:**
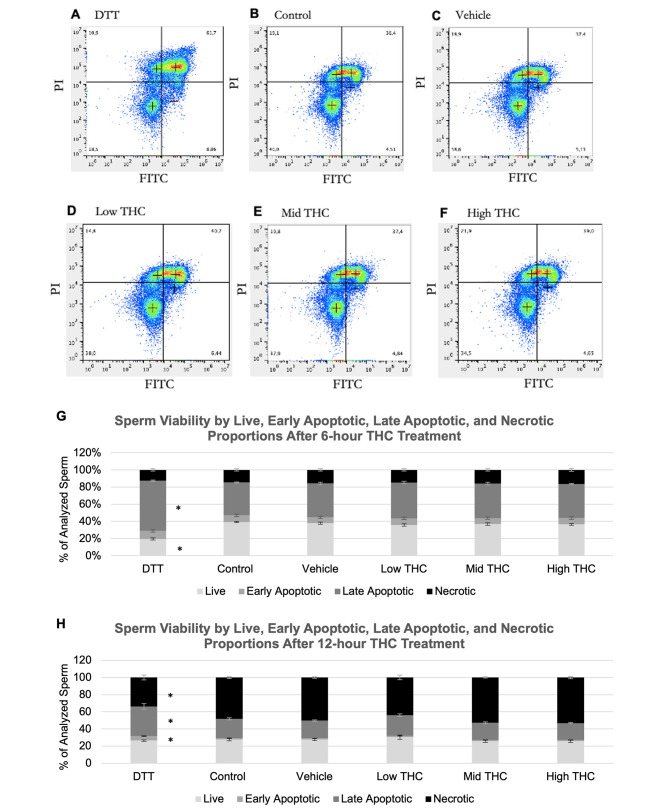
Sperm viability characteristics following THC-treatment. Effects of therapeutic (low THC) and recreational (mid and high THC) concentrations of THC on viability. Representative density plots of sperm in the (A) Dithiothreitriol (DTT) or apoptosis positive control group, (B) control group, (C) vehicle group (0.01% ethanol), (D) low THC group (0.032 µM THC), (E) mid THC group (0.32 µM THC), and (F) high THC group (3.2 µM THC). In each plot, the bottom left quadrant (PI and FITC negative) represent live sperm, the top left quadrant (PI positive, FITC negative) represent necrotic sperm, the top right quadrant (PI positive, FITC positive) represent late apoptotic sperm, and bottom right quadrant (PI negative, FITC positive) represent early apoptotic sperm. Average percentage of live, early apoptotic, late apoptotic, and necrotic proportions of sperm following (G) 6- and (H) 12-hours of treatment. PI, propidium iodide; FITC, Annexin V-FITC. Error bars represent ± SEM, n = 4. Asterisk (*) indicate p < 0.05

Sperm viability was measured from a total of four biological replicates following THC exposure for 6- and 12-hours, representing sperm from individual ejaculates from four bulls. Flow cytometry density plots indicated different quadrants for sperm subpopulations in each treatment group based on Annexin/PI staining patterns (Fig. 3A-F). In the 6-hour study, DTT-treated group had significantly less live sperm and more late apoptotic sperm compared to the control and vehicle (p < 0.05), whereas THC treatment did not affect viability (Fig. 3G). Similarly, in the 12-hour study, DTT induced early and late apoptosis (p < 0.05) but THC treatment did not alter viability (Fig. 3H).

### Effect of THC on sperm capacitation

**Fig. 4:**
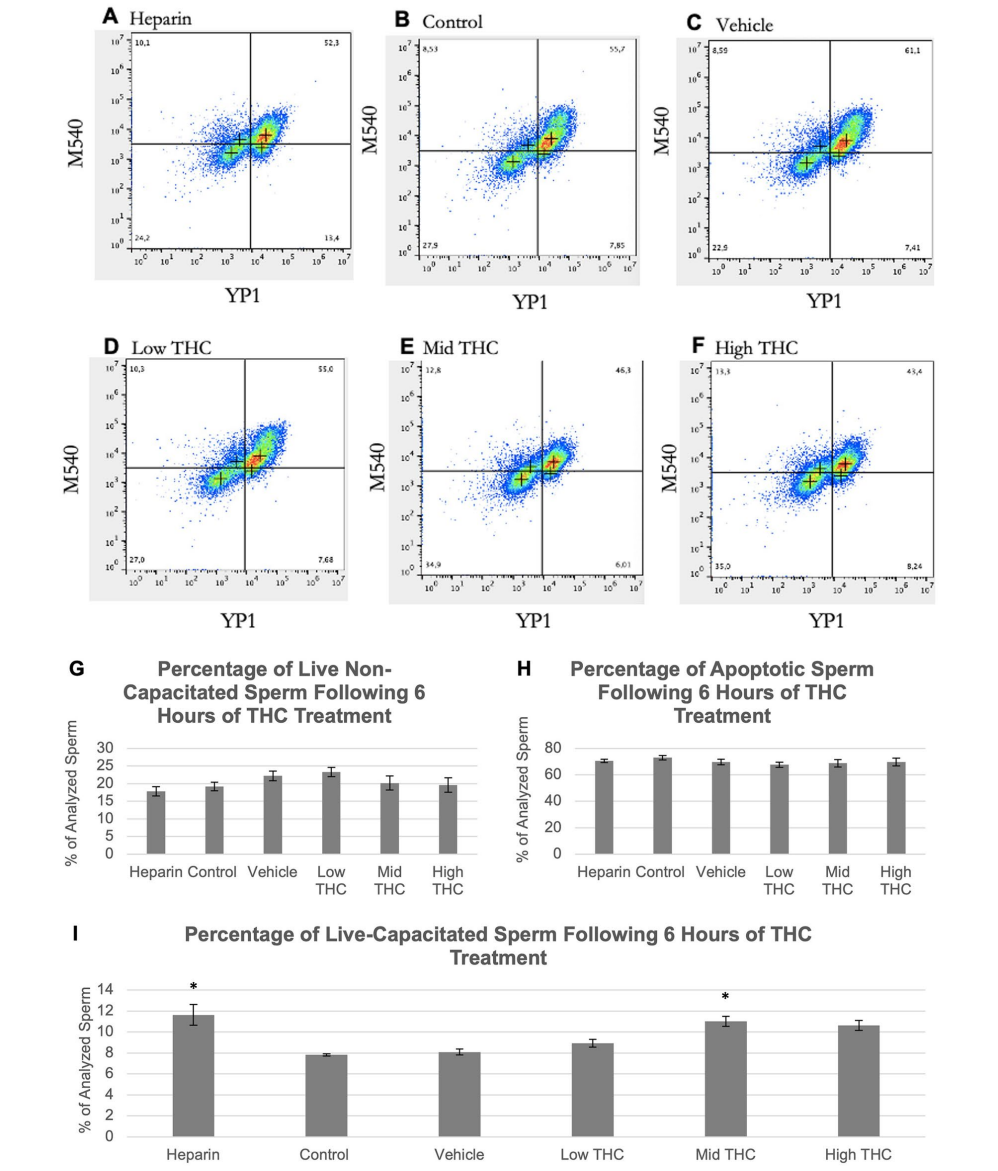
Sperm capacitation status following THC-treatment. Effects of therapeutic (low THC) and recreational (mid and high THC) concentrations of THC on capacitation. Representative density plots of sperm in the (A) Heparin or capacitation positive control group, (B) control group, (C) vehicle group (0.01% ethanol), (D) low THC group (0.032 µM THC), (E) mid THC group (0.32 µM THC), and (F) high THC group (3.2 µM THC). In each plot, the bottom left quadrant (Yo-Pro-1 and Merocyanine-540 negative) represent live and non-capacitating sperm, the top left quadrant (Yo-Pro-1 negative and Merocyanine-540 positive) represent live and capacitating sperm, and the two right quadrants (Yo-Pro-1 positive) represent apoptotic sperm. Average percentages of (G) live non-capacitating sperm, (H) apoptotic sperm, and (I) live capacitating sperm. YP1, Yo-Pro-1; M540, Merocyanine-540. Error bars represent ± SEM, n = 4. Asterisk (*) indicate p < 0.05

Sperm capacitation was measured from a total of four biological replicates following 6-hours of treatment, representing individual ejaculates from four bulls. Flow cytometry density plots showed the distribution of the sperm populations after incubation with M540/YP1 (Fig. 4A-F). Live/Non-capacitating and apoptotic proportions of sperm were unchanged by THC treatment (Fig. 4G-H), while the Mid-THC treated group exhibited significantly higher proportions of live-capacitating sperm in comparison to the control and vehicle (p < 0.05) (Fig. 4I). Heparin treatment also caused increased capacitation (p < 0.05) (Fig. 4I).

### Effect of THC on transcriptome

**Fig. 5:**
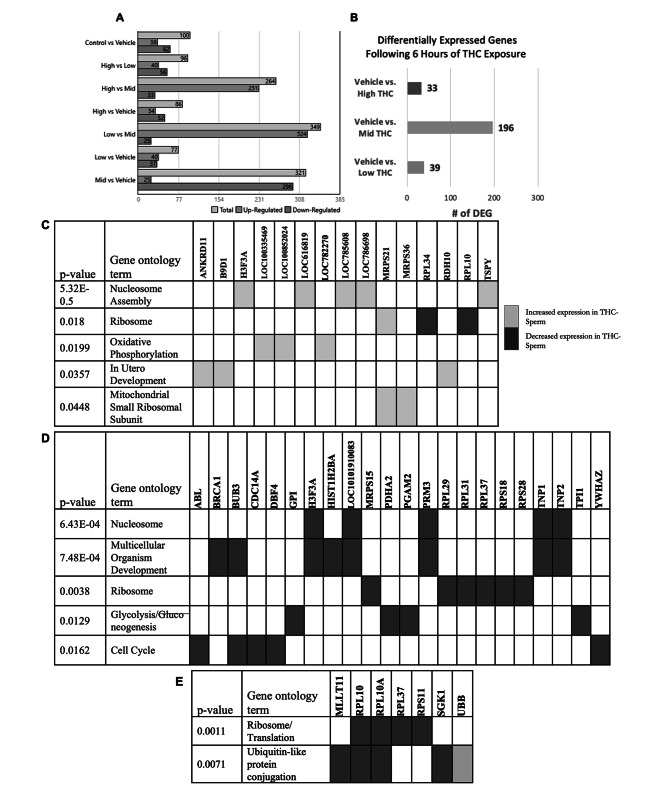
THC-induced changes to spermatozoal transcripts. Differentially expressed genes (DEGs) and gene ontology (GO) terms in sperm treated with therapeutic (low THC; 0.032 µM) and recreational (mid and high THC; 0.32 µM and 3.2 µM, respectively) concentrations of THC. (A) Total number of DEGs (upregulated and downregulated), (B) Filtered DEG to exclude unknown genes and DEG of the vehicle group, and enriched GO terms in the (C) low-THC DEGs, (D) mid-THC DEGs, and (E) high-THC DEGs. Dark and light boxes indicate decreased and increased expression, respectively, from THC treatment

To determine THC effects at the gene expression level, a transcriptome analysis was performed on two biological replicates of sperm. The first biological replicate consisted of RNA from sperm of a single bull, while the second biological replicate consisted of RNA from a pooled sample of sperm originating from three separate bulls (to mitigate individual variation). Differential gene expression analysis revealed 100 DEGs between sperm of the control and vehicle groups, 77 DEGs between the vehicle and low-THC, 321 between the vehicle and Mid-THC, and 86 between the vehicle and high-THC (Fig. 5A). After excluding unknown genes and genes that were differentially expressed between the control and vehicle groups, 39, 196, and 33 DEGs remained between the vehicle and low-THC, mid-THC, and high-THC sperm, respectively (Fig. 5B). There were 7 overlapping DEGs between the Low- and Mid-THC sperm, 14 overlapping between the Mid- and High-THC sperm, and four overlapping between the Low- and High-THC sperm. Three transcripts were detected at lower levels in THC-treated sperm compared to the vehicle-sperm, regardless of THC concentration; *ANKRD31 (Ankyrin repeat domain 31), B9D1 (B9 domain 1)*, and *ROPN1L (rhophilin associated tail protein 1-like).* Full DEG lists can be found in Supplementary Tables 1–3 in Additional File 4.

Secondary analysis of the DEG lists using DAVID 6.8 revealed several enriched gene ontology (GO) groups. Among the DEGs from the low-THC treatment sperm, GO terms relating to nucleosome assembly, ribosomes, oxidative phosphorylation, in utero development, and mitochondrial small ribosomal subunit were enriched (Fig. 5C). Among DEGs from the Mid-THC treated sperm, GO terms relating to the nucleosome, multicellular organism development, ribosomes, glycolysis/gluconeogenesis, and the cell cycle were enriched (Fig. 5D). Finally, GO terms ribosome/translation and ubiquitin-like protein conjugation were enriched based on the DEG list of High-THC treated sperm (Fig. 5E).

## Discussion

Sperm function can be influenced by exogenous compounds present in the body through several mechanisms. Previous literature has associated THC use in reproductive aged men with increased spermatid apoptosis, low sperm count, and teratozoospermia [29–34]. The presence of the ECS in the male reproductive tissues and its significant contributions to sperm activity warrant additional investigation into the potential effects of exocannabinoids, including the psychoactive phytocannabinoid THC, on sperm function using an in vitro model.

Regulated levels of eCBs are necessary for the development of sperm as well as mature sperm competence [21, 52, 53]. An existing gradient of eCBs decreasing from seminal plasma to oviductal and follicular fluid is suggested to spatio-temporally regulate sperm motility and capacitation by maintaining a suppressive effect on sperm until they are in closer proximity to the oviduct [54, 55]. Furthermore, stimulation of the cannabinoid receptors has generally been found to be inhibitory towards sperm activity [23, 24, 28].

In the present study, we studied the effects of physiologically relevant concentrations of THC (0.032µM, 0.32µM, and 3.2µM) representative of plasma levels of THC following therapeutic (low dose) and recreational (mid and high dose) cannabis use [41, 42, 56]. It should be noted that due to high lipophilicity, amount of THC in fatty tissues, such as the epididymis, following repeated THC exposure accumulates at levels up to 80 times higher than amounts in the brain and plasma [57]. As a result, the half-life of THC ranges from 6 min to as long as 22 h [58]. To our knowledge, there has only been one research group to quantify THC levels in the semen of cannabis users, who measured THC in semen 8–10 h following cannabis consumption and reported THC concentrations ranging from 0.87 to 0.97 ng/mL (equivalent to 2.7–3.1nM), below the range of concentrations used in our study [59]. However, since these findings are not likely to represent peak seminal fluid THC levels occurring sooner after consumption, we believe that the use of peak plasma concentration to approximate THC exposure was more appropriate.

Here, we assessed motility and morphology as parameters that have been traditionally used as indicators of sperm quality [35, 47]. We found no significant effects of THC on sperm motility or morphology, contrary to our initial hypothesis and previous literature documenting detrimental effects [23, 31, 41, 60]. It is likely that the lack of morphological changes after THC exposure in this study are a result of the use of mature sperm, whereas previous observations of teratozoospermia among cannabis users reflects the impact of THC during spermatogenesis. Interestingly, biphasic responses to THC in sperm have been recognized, where 2.5nM of methanandamide (Met-AEA), a stable analog of AEA, inhibited motility, but 0.25nM of met-AEA stimulated motility, suggesting that exocannabinoid effects on sperm are concentration-dependent [61].

Although the exact mechanisms are not fully understood, spermatozoal apoptosis involves the release of cytochrome C, caspase activation, and phosphatidylserine (PS) externalization, similar to somatic cell apoptosis [62–64]. Interestingly, the unique compartmentalization of sperm limit the availability cytoplasmic enzymes, which reduce the ability of the sperm to combat oxidative stressors [65]. Stimulation of the ECS is generally regarded to be pro-apoptotic as cannabinoid receptor stimulation causes ceramide accumulation leading to cellular stress responses and activation of the mitochondrial apoptotic pathway [66, 67]. Here, we assessed the effects of THC on sperm viability as a reflection of sperm physiology and potential ECS stimulation. We found no changes to viability resulting from THC exposure, in contrast to our hypothesis. Interestingly, Rossato et al. [23] report that 10µM of AEA, but not 0.1 or 1.0µM, eliminated sperm viability *in vitro.* Previous reports also implicate levels of THC beyond 30µM THC with the inhibition of mitochondrial membrane potential in sperm, a common indicator of apoptosis [68]. As a result, it is possible that apoptosis is only induced at concentrations substantially higher than the ones used in the present study.

The results of our motility, morphology, and apoptosis experiments may be explained by considering our use of cryopreserved bull sperm as an experimental model. Both extensive washing before and after the cryopreservation and rigorous selection process of bull sperm during commercial sperm cryopreservation leaves only the most robust sperm, which are reported to be more resistant to the effects of exocannabinoids [41]. On the other hand, cryopreservation necessitates removal of round somatic cells and seminal plasma, components which would otherwise be present during THC-sperm interactions and provide possible protective effects [68]. Lastly, the cryopreservation process is known to induce oxidative damage and apoptosis in sperm, which may have limited our potential to observe the effects of THC to viability [69, 70].

To further investigate THC-induced changes in sperm physiology, we examined capacitation, a necessary series of cellular and biochemical changes which allow sperm to propel through the female reproductive tract and prepare to fertilize the ovum [48, 71]. We observed that 6 h of 0.32µM of THC exposure, our mid-THC concentration, significantly increased the proportion of capacitating sperm compared to the sperm in the vehicle group, based on positive M540 and negative YP1 staining. Interstingly, sperm in the same group did not exhibit altered motility, which is typically observed in capacitating sperm through hypermotility. Previous research indicates conflicting evidence regarding the relationship between cannabinoids and capacitation. AEA and THC have been shown to inhibit the acrosome reaction, which is widely accepted as the culminating event of capacitation and is necessary for sperm fertilization competence [23, 41, 61]. On the other hand, 2-AG has been observed to increase the acrosome reaction in human sperm and met-AEA has been found to induce bull sperm capacitation [24, 72]. When intracellular calcium was measured as an indicator of capacitation, Gervasi and collegues [72] report only the two median concentrations of met-AEA elicited a significant change, similarly to the results we observed in the present study.

Our results indicate that THC can impact sperm physiology by promoting membrane disorder characteristic of capacitation. Although capacitation is a crucial aspect of sperm function, premature capacitation may unfavourably affect fertility by stimulating early hypermotility leading to exhaustion of sperm ATP, inability to reach the ovum, and premature death [54]. Moreover, premature acrosome reaction also impairs subsequent fertilization [48].

To our knowledge, this is the first study to explore and report effects of THC to transcripts in mature sperm. Cannabinoid receptor signalling in somatic cells can lead to downstream changes in gene expression [73–75]. However, a lack of transcriptional machinery limits transcriptional activity in sperm [76, 77]. Nonetheless, transcripts in mature sperm are known to be altered from cryopreservation and exposure to endocrine disrupting compounds, suggesting a mechanism for altered transcript levels [78–80].

We report over 200 DEGs in sperm exposed to THC, many relating to development, cellular metabolism, ribosomes and translation, and the nucleosome. It is noteworthy that the secondary gene ontology study performed found enrichment of DEGs relating to in utero development in the low-THC group, where all developmentally related genes had decreased expression, and multicellular organism development mid-THC group, where these genes had increased expression. We also report that all THC-treated sperm, regardless of concentration, shared enrichment in genes relating to ribosomes and translation, largely as a result from the differential expression of mitochondrial ribosomal proteins. Although sperm are generally thought to be translationally silent due to a lack of cytoplasmic ribosomes, Gur & Breitbart [81] described the presence of protein synthesized by mitochondrial ribosomes in mature sperm. Further research suggests that specific transcripts in mature sperm are actively translated by mitochondrial ribosomes during capacitation and the inhibition of mitochondrial translation is detrimental to capacitation, motility, and fertilization [81, 82]. These findings might provide insight on the considerable number of genes found to be decreased in sperm of the mid-THC group, which we also reported to have a significantly increased proportion of capacitating sperm. Since it is unlikely that changes in transcript abundance are due to transcriptional activity for reasons described previously, we speculate that the decrease in transcripts in this treatment group specifically was a result of transcript turnover stemming from increased mitochondrial ribosome activity during capacitation. Moreover, it is possible that the wide variety of transcriptomic changes observed in the presented study is a result of either translation dependent transcript turnover, accumulation, or altered stability. We can even speculate that, given the limited research on the mechanisms of transcript turnover in sperm, transcriptomic changes may be due to the inhibition of transcript degradation or decreased stability, both through mechanisms that have not yet been investigated in sperm. This is a limitation of this study, that warrants further experiments to confirm specific transcripts changes and to clarify the reason of these transcriptional changes.

In conclusion, our findings that THC induces capacitation provide evidence to support the growing body of facts suggesting that cannabinoids impact sperm physiology. To our knowledge, this is the first study to investigate the impacts of cannabinoids on transcripts in mature sperm. We report that THC can induce changes to sperm transcript levels, although additional validation studies should be performed prior to making functional conclusions. This study also utilized physiologically relevant concentrations of THC to mimic natural physiological responses as closely as possible in an in vitro model. Altogether, these findings justify further research on the cellular and molecular impacts of cannabinoids on sperm. In combination with previous work highlighting the detrimental effects of THC on sperm development and function, these results contribute to the growing body of research seeking to address the implications of cannabis use among reproductive aged men, the highest consumers of cannabis.

## Materials & methods

### Reagents

All media and chemicals were purchased from Sigma Aldrich (Oakville, ON, Canada) unless otherwise specified. THC stock was purchased as Dronabinol (Δ9-THC) from Toronto Research Chemicals (Toronto, ON, Canada).

### Semen preparation & treatment

Cryopreserved bull semen from bulls with known fertility were acquired from Semex (Guelph, ON, Canada). For each bull, 200uL of cryopreserved semen containing approximately 50 million sperm was thawed and washed using a discontinuous Percoll gradient described by Nguyen et al. [80]. The washed sperm was then split into one of five treatment groups containing 1mL of media each; HEPES/Sperm TALP supplemented with 0.3% bovine serum albumin (Control), 0.01% of ethanol diluted in the control group media (Vehicle), 3.2µM of THC diluted in the control group media (High-THC), 0.32 µM of THC made by 10-times serial dilution of High-THC media (Mid-THC), and 0.032µM of THC made by 10-times serial dilution of Mid-THC media (Low-THC). THC concentrations used in this study were meant to reflect plasma concentrations of THC following therapeutic and recreational cannabis use as described by Misner et al. [56]. Sperm were incubated at 38.5 °C with 5% CO_2_ until assessment.

### Effects of THC on motility and morphology

Sperm were retrieved after incubation for 6 and 12 h in the aforementioned conditions and centrifuged for 7 min at 2,500 RPM. Supernatant was removed and sperm pellet was resuspended in 50uL of warmed HEPES/Sperm TALP. For morphological assessment, 10uL of the resulting sperm suspension was spread onto a warmed glass slide using the pipette method [83] and air dried before being fixed overnight in a 3:1 methanol:acetic acid solution. Slides were then washed with water, stained with a Giemsa staining solution for 15 min, washed again, and evaluated under a Leica DMIRB microscope (Leica Microsystems; Richmond Hill, ON, Canada). Morphology of a minimum of 200 sperm per treatment group per biological replicate was manually assessed as previously described by Nguyen et al. [80].

For motility assessments, 10uL of sperm from resuspended pellet was placed onto a prewarmed Makler counting chamber and 90 s video recordings (25 frames per second) containing a minimum of 6 fields generated using the EPView camera and software (Olympus Life Sciences; Richmond Hill, ON, Canada). Recordings were used to manually evaluate the motility of a minimum of 100 sperm per treatment group per biological replicate as described by Nguyen et al. [80]. Both motility and morphology assessments were conducted by a single user and under blinded conditions.

### Effects of THC on sperm apoptosis and capacitation

Apoptosis was evaluated using the Annexin V-FITC Apoptosis Staining/Detection Kit consisting of binding buffer, propidium iodide (PI), and annexin V-FITC (FITC) (Abcam, Cambridge, MA, USA). Sperm were treated for 6 and 12 h and washed as previously described before being resuspended in 180uL of warmed binding buffer. A sixth treatment group containing 0.7µM of dithiotretriol (DTT) added 45 min before the end of the treatment period was used as a positive control for apoptosis. Three additional groups consisting of no stain, only FITC, and only PI controls required for flow cytometry compensation were included. In a dark room, 5uL of PI and FITC were added to each sample and incubated for 15 min before being assessed using the BD C6 Accuri Flow Cytometer (BD Biosciences, Mississauga, ON, Canada). FITC labels phosphatidylserine residues which accumulate on the outer leaflet of the lipid bilayer under apoptotic conditions while PI, a DNA dye, enters the cell upon membrane compromise during necrosis [84, 85]. FITC negative/PI negative staining represents the live sperm population, FITC positive/PI negative staining indicates early apoptosis, FITC positive/PI positive indicates late apoptosis, and FITC negative/PI positive indicates necrosis [86].

To assess capacitation, sperm were incubated for 6 h before being washed and resuspended in 180uL of warmed HEPES/Sperm TALP. Merocyanine-540 (M540) and Yo-Pro-1 (YP1) diluted in DMSO were added to sperm suspensions to achieve final staining concentrations of 50nM and 1.5µM, respectively. An additional group containing 10uL of 2U/mL heparin, a glycoaminoglycan known to induce capacitation in bovine sperm, was used as a positive control group [87, 88]. Three additional groups consisting of no stain, only M540, and only YP1 controls required for flow cytometry compensation were included. Samples were incubated in the dark before being assessed by flow cytometry. M540 intercalates into cell membranes with increasing membrane disorder while YP1, a DNA binding dye, enters early apoptotic cells with permeable membranes [89, 90]. Events that are YP1 positive indicate an apoptotic sperm, M540 positive/YP1 negative events represent live and capacitating sperm, and M540 negative/YP1 negative events represent the live and non-capacitating sperm [91].

Flow cytometry from both apoptosis and capacitation experiments were analysed using FlowJo v10 (BD Biosciences) using a minimum of 25,000 events per sample after gating to remove debris. Quadrants were determined based on no stain controls.

### Transcriptome analysis

Washed sperm from 400uL of cryopreserved semen were placed in 4mL of each of the treatment groups described previously for 6 h. Treated sperm pellets from two biological replicates were snap frozen and stored at -80 °C until total RNA extraction, using the Qiagen miRNeasy Micro RNA extraction kit according to manufacturer’s instructions (Qiagen, Toronto, ON, Canada). RNA samples from two biological replicates of bull sperm underwent a transcriptome analysis using a GeneChip Bovine Gene 1.0 ST Microarray (Thermo Fisher Scientific; Whitby, ON, Canada) at the David Braley Research Institute in Hamilton, Ontario, Canada. One biological replicate is representative of a single bull, while the second biological replicate consists of sperm pooled from three different bulls to mitigate individual variability.

Results of the transcriptome analysis were evaluated using Transcriptome Analysis Console (Thermo Fisher Scientific). Differentially expressed genes (DEG) were considered to be statistically significance if the fold change was greater than ± 2 and had a p-value of less than 0.05. The Database for Annotation, Visualization and Integrated Discovery (DAVID 6.8) was used to perform a secondary gene ontology analysis.

### Statistics

Statistical analysis was performed on GraphPad Prism 6 using data from at least 3 biological replicates. All data sets were subjected to the Kolmogorov-Smirnov test of normality. Normally distributed data sets were analyzed using one-way analysis of variance (ANOVA) and non-normally distributed data sets were analyzed using the Kruskal–Wallis test. Tukey’s *post-hoc* test was used to determine statistical significance between the treatment groups.

## Electronic supplementary material

Below is the link to the electronic supplementary material.

Supplementary Material 1

Supplementary Material 2

Supplementary Material 3

Supplementary Material 4

## Data Availability

The datasets used and/or analysed during the current study are available from the corresponding author on reasonable request.
